# Workplace Digital Health Is Associated with Improved Cardiovascular Risk Factors in a Frequency-Dependent Fashion: A Large Prospective Observational Cohort Study

**DOI:** 10.1371/journal.pone.0152657

**Published:** 2016-04-19

**Authors:** R. Jay Widmer, Thomas G. Allison, Brendie Keane, Anthony Dallas, Kent R. Bailey, Lilach O. Lerman, Amir Lerman

**Affiliations:** 1 Division of Cardiovascular Diseases, Department of Internal Medicine, Mayo Clinic and College of Medicine, Rochester, MN 55905, United States of America; 2 CareHere, Inc., Nashville, TN, United States of America; 3 Division of Nephrology and Hypertension, Department of Internal Medicine, Mayo Clinic and College of Medicine, Rochester, MN 55905, United States of America; 4 Division of Biomedical Statistics and Informatics, Mayo Clinic and College of Medicine, Rochester, MN, 55905, United States of America; Vanderbilt University, UNITED STATES

## Abstract

Cardiovascular disease (CVD) is the leading cause of morbidity and mortality in the US. Emerging employer-sponsored work health programs (WHP) and Digital Health Intervention (DHI) provide monitoring and guidance based on participants’ health risk assessments, but with uncertain success. DHI–mobile technology including online and smartphone interventions–has previously been found to be beneficial in reducing CVD outcomes and risk factors, however its use and efficacy in a large, multisite, primary prevention cohort has not been described to date. We analyzed usage of DHI and change in intermediate markers of CVD over the course of one year in 30,974 participants of a WHP across 81 organizations in 42 states between 2011 and 2014, stratified by participation log-ins categorized as no (n = 14,173), very low (<12/yr, n = 12,260), monthly (n = 3,360), weekly (n = 651), or semi-weekly (at least twice per week). We assessed changes in weight, waist circumference, body mass index (BMI), blood pressure, lipids, and glucose at one year, as a function of participation level. We utilized a Poisson regression model to analyze variables associated with increased participation. Those with the highest level of participation were slightly, but significantly (p<0.0001), older (48.3±11.2 yrs) than non-participants (47.7±12.2 yr) and more likely to be females (63.7% vs 37.3% p<0.0001). Significant improvements in weight loss were demonstrated with every increasing level of DHI usage with the largest being in the semi-weekly group (-3.39±1.06 lbs; p = 0.0013 for difference from weekly). Regression analyses demonstrated that greater participation in the DHI (measured by log-ins) was significantly associated with older age (p<0.001), female sex (p<0.001), and Hispanic ethnicity (p<0.001). The current study demonstrates the success of DHI in a large, community cohort to modestly reduce CVD risk factors in individuals with high participation rate. Furthermore, participants previously underrepresented in WHPs (females and Hispanics) and those with an increased number of CVD risk factors including age and elevated BMI show increased adherence to DHI, supporting the use of this low-cost intervention to improve CVD health.

## Introduction

Cardiovascular disease (CVD) is the primary contributor to increased morbidity, mortality, and rising health care associated costs in the United States. Recent estimates attribute over one in every three deaths to CVD[1,2], and over 90% of CVD morbidity and mortality to preventable risk factors[3]. Despite extensive knowledge of the risk factors and aggressive measures to correct lifestyle habits, CVD continues to be highly prevalent in Western Society.

Work health programs (WHPs) have successfully reduced healthcare costs [4] and improved lifestyle behaviors such as poor diet and lack of physical activity, leading to improved levels of risk factors for CVD [5,6]. These WHPs have increasingly incorporated digital health interventions (DHI)–such as online and mobile platforms used in managing health and disease–in an effort to widely disseminate educational aspects while simultaneously gathering data and using it as feedback for the employees to monitor and improve their own health. We have recently demonstrated reductions in CV risk factors and Framingham Risk Scores (FRS) at a single employer using a DHI as a WHP after only 90 days [7]; however longer follow up and larger cohorts are needed to fully quantify these effects. This is especially pertinent now that data are emerging indicating an overall cardiovascular morbidity and mortality benefit seen with DHI [8,9]. The current study was designed to extend our previous observations, and evaluate potential predictors of use, and the success associated with use, of a WHP DHI.

Thus, we prospectively followed a similar, but larger, cohort incorporating multiple employers in an effort to 1) validate this particular WHP DHI in a larger population and 2) study potential factors associated with patterns in DHI adherence and associated success in reducing CVD risk factors.

## Materials and Methods

### The Digital Health Intervention (DHI)

**Fig 1:**
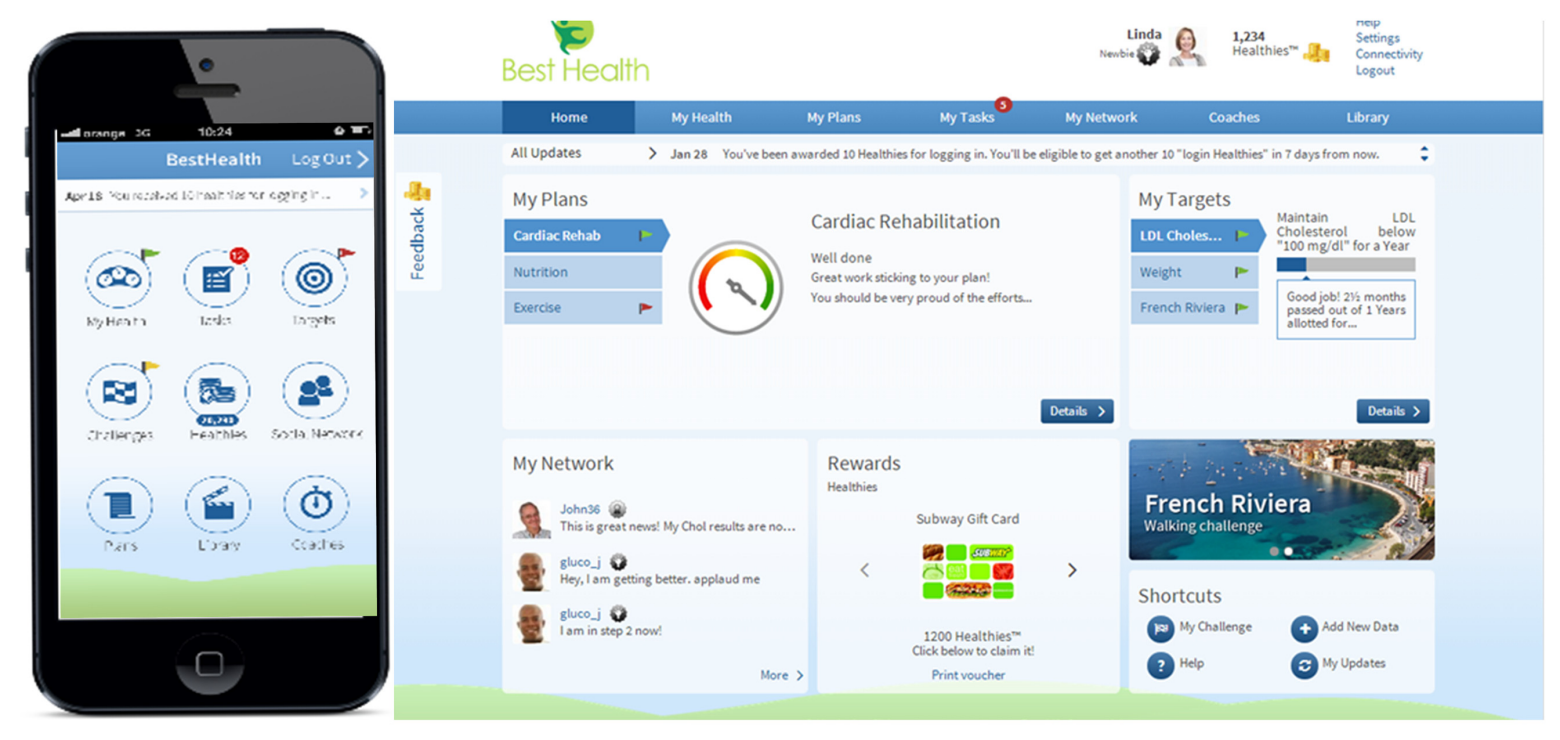
Smartphone (left) and online (right) versions of the digital health intervention. The online version (right) shows the “Lifestyle” dashboard comprised of CVD risk factors such as smoking status, physical activity, dietary habits, and medication adherence. Patients are able to learn about these habits individually, and note their progress over time. The smartphone version (left) demonstrates the home page of the mobile platform allowing patients to navigate to any portion of the program and enter their own data.

The DHI, previously described [7], is an online and smartphone-based portal which tracks and logs, contains educational, non-commercial material, and gives the user actionable tasks to improve their health (Fig 1). Participants are instructed on uploading baseline information, then tracking and inserting their own health information as they progress through the program. Individualized care plans are offered based on medical diagnoses and comorbidities. It provides user-friendly and interactive access to health status information, tasks, targets, and plans that encourage the adoption and maintenance of a healthier lifestyle for improved wellness. Reminders to complete tasks may be received via email or SMS text messaging. The software is linked with an electronic health record affiliated with CareHere, LLC and with the respective employer, to provide continuity of information and healthcare. This record is able to capture ICD codes and medical diagnoses, medication information, demographic information, as well as vital signs/lab information. This particular program integrated with the workplace health program electronic medical record (EMR), and data gathered are a combination of patient reported and EMR-derived information.

### Employee Recruitment and Study Parameters

Between 2011 and 2014 CareHere, LLC (Nashville, TN) created and implemented an incentive plan for employees to improve their health across 81 employers in 42 states encompassing 30,974 employees in separate governmental, white collar, blue collar, and other occupations. CareHere, LLC’s onsite clinic vendor managed the individual programs and tracked results both manually and with the Online CareHere Connect PHA (Personal Health Assistant) designed and produced by Healarium, Inc (Dallas, TX). As described previously [7], all employees enrolled in the employer-sponsored health insurance program were offered the opportunity to participate as a normal part of their insurance process, to complete biometric screening. The DHI software was similar in that they covered basics of CVD prevention, but were individualized for each employer, across the entire patient population as it was conceived and designed by CareHere. Delivery methods and interventions were also similar, and did not vary by employer. Employees were given the option to “opt out” of the digital health component of the program upon the initial intake, but were not consented at the time of entry, as deidentified data were used in the analyses. The study and consent process were approved by the IRB of Mayo Clinic. The study was conducted according to the principles expressed in the Declaration of Helsinki.

Standard laboratory blood tests including fasting lipid panels and serum glucose values were assessed at baseline and every 90 days for one year for those participating and were paid for through the WHP and insurance. The patients’ primary health care providers assessed blood pressure, height, weight, and the health behavior questionnaires at baseline and every 90 days in a standard fashion. Employees were asked to follow up with a healthcare provider every 90 days for the duration of their WHP (at least one year) to review results and see if all health benchmarks set by the employer were met.

All participants analyzed were offered use of the program. Participants were categorized according to DHI use as no (n = 14,173), very low (<12/yr, n = 12,260), monthly (n = 3,360), weekly (n = 651), or semi-weekly (greater than once per week; n-265) log-ins based on prior work [7] showing similar usage in a smaller but congruent population. Baseline demographic data were collected including age, gender, ethnicity, job type, and state of employment. Follow up assessment every 90 days for at least one year consisted of a collection of the baseline parameters (weight, blood pressure, lipids, glucose) in a similar fashion. Data were abstracted and collected through electronic medical records associated with the respective employer. De-identified data were transmitted through Healarium, Inc. to investigators at Mayo Clinic for a comprehensive data analysis at the completion of the program.

### Statistical Analysis

For binary data, percentages were calculated and chi-square tests were used to test group differences. For normally distributed continuous data, means ± standard deviations were calculated and presented. To examine the effect of increased usage on CV risk factors, we fit a general linear model analyzing the incremental changes in CVD risk factor surrogates as a function of increased log-ins, reporting the adjusted changes in CVD risk factors as well as the incremental difference between usage groups, adjusting for age, sex, employer, and ethnicity, with or without adjustment for other baseline factors. In addition, we considered the scale (0 = none, 1 = some, 2 = monthly, 3 = weekly, 4 = biweekly) as a linear scale, in order to detect any trend. In this model, we treated employer as a fixed effect, which removes employer as a confounding effect between usage and outcomes, and also removes any intra-employer correlation from the y-variable. In order to assess predictors of DHI usage, we performed a fixed-effects Poisson regression model of cumulative log ins to identify variables associated with increased participation including age, gender, ethnicity, employer, baseline weight, baseline hypertensive status, baseline glucose, and baseline lipid status. For this model age was evaluated both as linear and as a quadratic function. A conventional alpha level of 0.05 was used in all models and for all data to determine statistical significance. No data from the index publication were used in these analyses [7].

## Results and Discussion

The baseline demographics of the participants and non-participants are described in Table 1. Those studied were predominantly white (72%), worked in governmental jobs (79%), and resided in 42 different states in the US. Employers were to a large extent, though not entirely, nested within states, such that adjusting for employer effectively removed the need to adjust for state. Those who utilized the program were older, more likely to be female, were more obese, had worse lipid profiles, and higher glucose levels. Participation in the program was largely via the web-based portal (88%) compared to iOS (7%), and Android (5%) use. The mean number of medications was 1.5±2.7, and the mean number of ICD-9 codes listed on each participant’s chart and assigned per participant was 10.5±8.5. The range of log-ins for users was 0 to 338 with low-participants averaging 5.4±5.8 log-ins yearly and high-participant users averaging 58.5±41.36 yearly. Generally there was a decline in log-ins throughout the year, but no significant differences.

**Table 1:** Baseline parameters of Non-participants, very low-use participants (those who logged in less than 12 times per year), monthly participants, weekly participants, and semi-weekly (twice per week) participants of the DHI-based WHP.

Category	Non-Participant (n = 14,173)	Very Low Use (<12/yr; n = 12,260)	Monthly Use (n = 3,630)	Weekly Use (n = 651)	Semi-weekly Use (n = 260)	Total (n = 30,974)
Age (yrs)	47.7±12.2	48.4±11.2	47.8±11.3	48.9±11.0	49.9±11.1	48.1±11.7
Sex (Male)	7,034 (49.7%)	4,459 (36.4%)	1,295 (35.7%)	244 (37.5%)	104 (40%)	13,136 (42.4%)
White	9,625 (67.9%)	9,216 (75.2%)	2,766 (76.2%)	481 (73.9%)	208 (80%)	22,296 (72.0%)
Government Workers	11,798 (83.2%)	9,407 (76.7%)	2,697 (74.3)	472 (72.5%)	185 (71.2%)	24,559 (79.4%)
White Collar	695 (4.9%)	1,021 (8.3%)	271 (7.5%)	35 (5.4%)	20 (7.7%)	2,042 (6.6%)
Blue Collar	1,214 (8.6%)	1,193 (9.7%)	447 (12.3%)	96 (14.7%)	46 (17.7%)	2,996 (9.7%)
Weight (lbs)	196.3±50.0	198.4±51.1	199.3±49.4	194.2±50.9	195.3±49.5	197.4±50.4
Waist Circ (in)	36.8±6.2	37.8±6.5	38.3±6.4	37.6±6.4	37.6±6.5	37.4±6.4
BMI (kg/m^2^)	30.1±6.7	31.1±7.1	31.3±6.9	30.4±6.9	30.4±6.5	30.7±6.9
Systolic BP (mmHg)	123.6±14.5	123.4±14.1	122.8±13.3	123.5±13.1	121.5±13.2	123.4±14.2
Diastolic BP (mmHg)	77.7±9.7	77.8±9.2	77.7±8.9	78.0±9.0	77.2±9.2	77.8±9.4
Triglycerides (mg/dL)	130.8±80.2	140.1±82.7	135.5±74.3	134.2±76.6	132.0±80.3	134.7±80.6
LDL (mg/dL)	111.2±32.0	112.1±32.7	110.7±32.0	112.7±31.8	108.0±29.9	111.5±32.3
HDL (mg/dL)	52.2±15.0	51.8±15.1	51.1±14.5	51.9±15.0	52.8±15.7	52.0±15.0
Glucose (mg/dL)	98.8±27.4	101.1±29.9	100.4±26.7	103.2±32.9	98.4±27.5	99.9±28.5
HbA1C (%)	6.6±1.6	6.5±1.6	6.3±1.5	6.4±1.5	6.4±1.5	6.5±1.6

Changes over one year in CVD risk factors are highlighted in Table 2 showing improvements on average in nearly all CVD risk factors among those who had any level of participation. Notably, those with a higher level of participation had significantly greater reductions in weight (-5.24 lbs; p<0.001) and improvements in HDL cholesterol (+0.90 mg/dL) compared to those with either no or low-participation, respectively. Furthermore, we note incremental gains in weight loss and HDL increases in those with higher DHI usage frequency (Table 2).

**Table 2:** Changes from baseline in CVD risk factors after one year among non-participants, very low-use participants (those who logged in less than 12 times per year), monthly participants, weekly participants, and semi-weekly (twice per week) participants in the DHI-based WHP. (#—When treated as a scale, the association between frequency and HDL increase was highly significant (p = 0.0082). @—When treated as a scale, the association of frequency with glucose change was negative, with a p-value of 0.083.).

Frequency Of usage	Adjusted mean weight change	Delta from increased frequency	Adjusted mean systolic blood pressure change	Delta from increased frequency	Adjusted mean HDL change#	Delta from increased frequency	Adjusted mean glucose change@	Delta from increased frequency
None	+2.34		-0.99		-0.64		-2.61	
		-0.87±0.32 (p = 0.007)		-0.05±0.36 (p = 0.89)		0.11±0.28 (p = 0.69)		-0.09±0.58 (p = 0.88)
< monthly	+1.47		-1.04		-0.53		-2.70	
		-1.71±0.37 (p<0.001)		-0.39±0.45 (p = 0.39)		0.45±0.36 (p = 0.21)		-1.67±0.74 (p = 0.025)
monthly	-0.25		-1.43		-0.08		-4.37	
		-1.59±0.67 (p = 0.018)		-1.13±0.88 (p = 0.20)		0.98±0.69 (p = 0.15)		0.22±1.42 (p = 0.88)
weekly	-1.83		-2.56		+0.90		-4.15	
		-3.39±1.06 (p = 0.0013)		+2.06±1.44 (p = 0.15)		-0.01±1.12 (p = 0.99)		2.21±2.36 (p = 0.35)
Bi-weekly	-5.24		-0.49		+0.89		-1.94	

**Fig 2:**
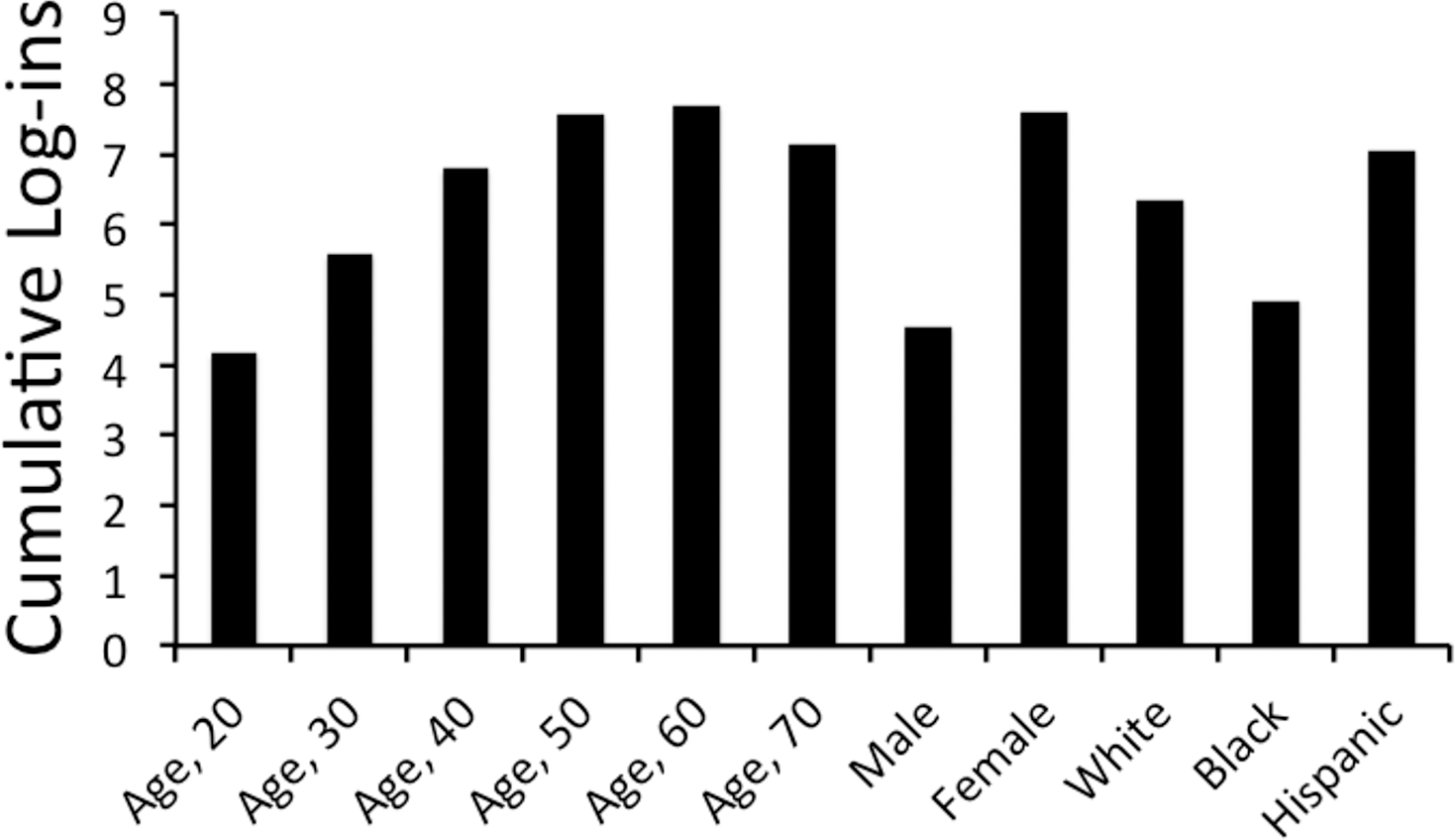
Predicted means of log-ins adjusted for age, gender, job type, employer, ethnicity, state, baseline weight, baseline systolic blood pressure, baseline glucose, and baseline lipid status.

A Poisson regression model was used to investigate the association between total logins and gender, age, and ethnicity. We also adjusted for age, gender, job type, employer, ethnicity, state, baseline weight, baseline systolic blood pressure, baseline glucose, and baseline lipid status. From this, we note women, on average, tended to have 3.07 more total log-ins (p<0.001) at one year compared to men (Fig 2). Similarly, we found significant associations between older age, Hispanic ethnicities, and total logins as described in Table 3. Of note, employment type, white or African American ethnicity, lipid status, glucose had no predictive effect in terms of use of the program.

**Table 3:** Poisson regression model. Here we present estimates and p-values outlining the association between baseline parameters and cumulative log-ins over one year. Patients who were older, female, of multiple or Hispanic ethnicities, and had increased waist circumference at baseline were more likely to participate throughout the year. (*p-value meets significance at a value <0.05).

Category	Estimate Size	Standard Error	P-Value
Age	0.0695	0.0017	<0.001*
Ethnicity (White)	0.2955	0.0085	<0.001
Ethnicity (Hispanic)	0.3387	0.0141	<0.001*
Ethnicity (Black)	0.0569	0.0130	<0.001
Ethnicity (other)	0.00	0.00	
Gender (Female)	0.4325	0.0055	<0.001*
Gender (Male)	0.00	0.00	
Weight (lbs)	0.0027	0.0001	<0.001
Systolic BP (mmHg)	-0.0005	0.0002	<0.0093

## Conclusions

The current results demonstrate that an employer-initiated health program utilizing digital health across multiple employers across the US effectively reduces CVD risk factors over one year of use. Participants using the DHI with greater frequency have more pronounced weight loss and improvements in blood pressure as well as in lipids. Furthermore, we report a significant association between DHI use and the female gender and older participants. The current study, for the first time, demonstrates the feasibility and efficacy of DHI in the reduction of CVD risk factors in a large, diverse cohort of willing participants across the US, and furthermore, supports the use of this potentially cost-effective tool for the reduction of CVD risk.

This work extends our prior results showing an improvement in FRS in employees using DHI, and serves to validate that a large-scale WHP can be effective in reducing CVD risk factors across employees from multiple employers. Extending our previous work which outlined a comprehensive improvement in CVD risk factors with a 90-day DHI-based WHP [7] at a single site in only 500 participants, these results show a beneficial effect of DHI on CV risk factors such as weight loss in diverse work and geographical environments for more than 30,000 participants over the course of one year. Previous WHP interventions have shown a positive impact on CVD risk factors over a shorter duration of time [10]. However, integration of DHIs has failed to show overwhelmingly a reduction in CVD risk factors, or create cost savings for the sponsoring employer [11]. Certainly, prior WHPs utilizing a DHI platform were challenging in terms of increasing the scale of use while maintaining portability, and the factors which predict use have remained elusive until now.

The current study was not designed to evaluate cost-savings or hard CVD outcomes; however, the large population did allow for evaluation of the relationship between frequency of use and the effect of this DHI across multiple employers. Our data show a frequency dependent effect of the WHP DHI across this large cohort, with only a modest use (an average of twice monthly) associated with improved weight loss and any use associated with improved lipids and blood pressure control compared to non-users. The reduction in CVD risk factors seen in this large cohort mirrors the improvements in risk factors we describe in a recent systematic review and meta-analysis on DHI for CVD prevention [8] which reports an overall benefit on CVD outcomes with digital health use predominantly in secondary prevention settings. While seemingly diminutive, these modest gains in secondary CVD intermediates can have a profound clinical implication as even weight loss of 2–5% of baseline body weight over one year is associated with a stark reduction in progression toward diabetes. These same investigators note similar observations are noted for changes in blood pressure and lipids [12]. Our patient population is quite similar to those in the Look AHEAD registry, and would certainly be targeted by interventions such as DHI–and certainly more likely to be successful with increased use. What is more, the current database is substantially larger than in previous analyses and most of the studies were a follow up of less than one year. According to these data such a non-intrusive, inexpensive, ubiquitous intervention conveniently situated in the workplace has a potential to powerfully impact CVD risk factor control, particularly among females and older participations–both deemed to be neglected in the field of CVD prevention [13]. Certainly, the potential applicable patient population is broad, as demonstrated across this cohort throughout the US in multiple work settings and with a reasonably robust response in non-white participants. Certainly the ability to yield such benefit in a cost-efficient manner by which the instrument can be adjusted based on the targeted population to include incentives and social media–two factors absent in this study–could have an even more widespread impact. Furthermore, skepticism has waned in the past months with increased Medicare/Medicaid coverage for chronic conditions through these means [14] and relaxation of federal oversight in matters pertaining to DHI [15]. Clearly, more data are needed to ascertain the mechanisms behind these trends, and to better individualize these therapies to incorporate the non-users.

Previous works in the field of WHP and DHI have encompassed single sites with no more than a few hundred employees over the course of a few months. A recent randomized controlled trial (RCT) out of Denmark shows generally positive benefits on physical activity habits as well as blood pressure over a 10-week period [16] harnessing the power of company emails. Prospective data from a study on 368 patients followed for one year demonstrated a 17.9% relative CVD risk reduction in patients at high risk for CVD (>20%) at baseline, yet final participation/response rate was only 50% [5]. Another study randomized overweight employees to a DHI (phone calls, emails, internet) versus usual care, but were only able to demonstrate modest reductions in weight, lipids, and aerobic fitness levels out to two years [17]. These studies show positive benefits of WHP DHIs, and are in accord with data from our group showing a reduction in FRS in a single employer cohort after 90 days [7]. Nevertheless, none of the aforementioned studies followed a large cohort of patients up to one year across multiple employment sites similar to the data presented in the current study. Furthermore, the positive response in non-whites such as Hispanics and mixed race participants is certainly encouraging despite comprising less than ten percent of the study population. In fact, of the 53 million Hispanics in America, their rate of cell phone and internet adoption surpasses that of most ethnic groups with 86% owning a smartphone and 76% having access to the internet through some device [18]. While the relative uptake in digital/mobile health technologies has not been overwhelming in these populations, these data show that efforts should be made to create means by which we can engage and treat these populations which certainly have their own set of increased CVD risk factors.

Previous work has outlined unsuccessful WHP DHIs in highly educated individuals pointing out the importance of implementation dose and delivery method in determining success of these types of interventions [19]. Indeed, we show that there is a frequency-dependent effect of this DHI, and those previously thought to benefit least from DHI–females, older participants–actually exhibited a more robust utilization and subsequent CVD risk factor improvement according to these data. Success in losing weight was also highly dependent upon older age and increased use of the program. We also demonstrated that those with more CVD risk including increased waist circumference will use the system with greater frequency, further addressing unmet needs in the digital health and mobile health fields of designing therapies for those with the greatest potential to benefit. The ubiquitous success of this DHI points to the potential widespread benefit of these therapies if carefully designed and implemented, and furthermore establishes the notion that there is a dose-dependent effect of WHP DHIs.

While this work shows beneficial results, it does contain challenges inherent to a large prospective cohort study. Primarily, its non-randomized design and lack of a separate control group restricts its implications, yet opens up the possibility for future randomized trials carefully designed to study which specific facets of the program are most responsible for the positive benefits. Despite heterogeneity in some of the figures, it is estimated that workplace DHI increased WHP engagement 10–50% across this sample, thereby negating some potential selection bias as such a broad mix of employees are rarely included in these types of analyses. Additionally, we know from our prior observational DHI and cardiac rehabilitation studies that approximately 50% of those approached for these types of studies agree to participate [18].” Moreover, we did include a variety of employees in terms of geographic distribution, job type, ethnicity, gender, and age. Unfortunately we were unable to accurately characterize any changes in medications during the course of the study despite the patients being seen by providers on a regular basis while in the program. Moreover, utilizing survey data in an historical fashion always will have discrepancies in provider data and patient provided data. Nevertheless, these data can contribute to the overall knowledge-base in implementing some of these digital health programs in the workplace and potentially population-based setting in an effective and cost-effective fashion.

In conclusion, we demonstrate for the first time a widely-distributed, DHI-based WHP is associated with improved weight loss, blood pressure control, and lipid profiles in a frequency-dependent fashion. Furthermore, we describe increased use among females and older participants in this employee-based population, irrespective of ethnicity or employer type. These results show promise in implementing employer-sponsored programs in an effort to ameliorate the nation-wide burden of CVD, and provide insight into the demographics behind those who are successful with such programs.
